# All tangled up: interactions of the fibrinolytic and innate immune systems

**DOI:** 10.3389/fmed.2023.1212201

**Published:** 2023-06-02

**Authors:** Claire S. Whyte

**Affiliations:** Aberdeen Cardiovascular and Diabetes Centre, The Institute of Medical Sciences, School of Medicine, Medical Sciences and Nutrition, University of Aberdeen, Aberdeen, United Kingdom

**Keywords:** fibrinolysis, innate immune, infection, plasminogen, thrombosis

## Abstract

The hemostatic and innate immune system are intertwined processes. Inflammation within the vasculature promotes thrombus development, whilst fibrin forms part of the innate immune response to trap invading pathogens. The awareness of these interlinked process has resulted in the coining of the terms “thromboinflammation” and “immunothrombosis.” Once a thrombus is formed it is up to the fibrinolytic system to resolve these clots and remove them from the vasculature. Immune cells contain an arsenal of fibrinolytic regulators and plasmin, the central fibrinolytic enzyme. The fibrinolytic proteins in turn have diverse roles in immunoregulation. Here, the intricate relationship between the fibrinolytic and innate immune system will be discussed.

## Introduction

Over the last two decades there is increasing awareness of immunothrombosis, where components of the immune system promote coagulation to limit the action of invading pathogens (1). Whilst thromboinflammation describes the inflammatory process induced by pathogens leading to platelet-neutrophil and platelet-monocyte interactions and endothelial dysfunction that promote a prothrombotic environment (2, 3). Activation of monocytes and neutrophils induces release of tissue factor (TF) promoting the extrinsic coagulation pathway, whilst intrinsic coagulation is triggered by binding of factor XII (FXII) to neutrophils (4). Additionally, activated neutrophils degranulate and expel their nuclear and cytoplasmic content to form neutrophil extracellular traps (NETs) during the neutrophil death process, NETosis (4). NETs act as a surface for assembly of procoagulant proteins including TF, FXII and von Willebrand factor (5). Furthermore, released neutrophil elastase cleaves tissue factor pathway inhibitor, thereby dampening the anticoagulant effect and contributing to fibrin persistence (6). The fibrinolytic system is responsible for limiting ongoing fibrin formation and degrading the fibrin meshwork to resolve thrombi.

## Fibrinolysis

**FIGURE 1:**
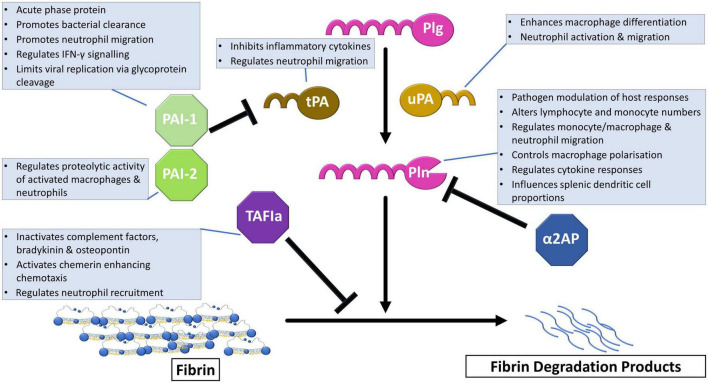
The fibrinolytic system in immune regulation. Plasminogen (Plg) is converted to the active enzyme plasmin (Pln) after cleavage by tissue plasminogen activator (tPA) or urokinase (uPA). This step is regulated by plasminogen activator inhibitor-1 (PAI-1), which is the primary physiological inhibitor, and plasminogen activator-2 (PAI-2). The active enzyme, plasmin, cleaves crosslinked fibrin into fibrin degradation products that can be cleared from the circulation. Alpha2-antiplasmin (α2AP) directly inhibits plasmin by forming a non-covalent complex. Activated thrombin activatable fibrinolysis inhibitor (TAFIa) exerts its effects by removal of C-terminal lysine required for plasminogen binding to fibrin. Boxes detail functions of the fibrinolytic proteins in immune regulation.

Plasmin, the central enzyme responsible for fibrin degradation is formed after cleavage of Arg_561_-Val_562_ of the zymogen form, plasminogen, via plasminogen activators (Figure 1). The primary physiological activators are tissue plasminogen activator (tPA) and urokinase (uPA). Efficient tPA-mediated plasminogen activation requires binding of both proteins to fibrin or cellular surfaces. uPA-mediated activation can occur in solution, although it can be localized to cellular surfaces via urokinase plasminogen activator receptor (uPAR) (7). The fibrinolytic system is normally tightly regulated by various inhibitors. Plasminogen activation is primarily regulated by plasminogen activator inhibitor-1 (PAI-1) which forms a 1:1 complex with the activators (8). Plasminogen activator inhibitor-2 (PAI-2) is not as efficient an inhibitor as PAI-1 but does function in uPA-mediated extracellular activity (9). The principal plasmin inhibitor is the serine protease inhibitor (SERPIN), α2-antiplasmin (α2AP) which forms a non-covalent complex with the active enzyme (10). Crosslinking of α2AP to fibrin by active transglutaminase factor XIII (FXIIIa) enhances the ability of this SERPIN to inhibit plasmin (11). Thrombin activatable fibrinolysis inhibitor (TAFI) further acts as a fibrinolytic break by removing C-terminal lysines from fibrin which are required for the binding of plasminogen and tPA.

## Pathogen hijacking of the fibrinolytic system

Invading pathogens take advantage of the fibrinolytic system, activating plasminogen in order to remove the confines of fibrin and extracellular matrix barriers and to evade the innate immune system (12). Indeed, certain strains of bacteria can produce plasminogen activators. Beta hemolytic strains of *Streptococci* possess streptokinase which induces non-proteolytic plasminogen activation by causing a conformational change that exposes the catalytic site and this complex can hydrolytically activate other plasminogen molecules (13). *Staphylococcus aureus* produces staphylokinase which also non-proteolytically activates plasminogen by forming a complex which generates plasmin (14). Staphylokinase is considered to be fibrin specific and in the absence of fibrin it is susceptible to inhibition by α2AP (15). Whilst *Yersinia pestis*, are able to proteolytically activate plasminogen and scuPA by the membrane protein Pla (16). Plasminogen contributes to lethality of *Y. pestis*, promoting spread of the bacteria and dampening immune cell recruitment to sites of infection [reviewed in (17)].

Additionally, a plethora of plasminogen binding proteins (e.g. α-enolase, glyceraldehyde-3-phosphate dehydrogenase (GAPDH) and PAM) exist on bacteria, fungal pathogens, protozoan and helminth parasites (12, 18, 19). Bacteria utilize plasminogen to remove fibrin barriers and enable invasion through extracellular matrices both directly and indirectly by activating matrix metalloproteases (17, 19–21). Additionally, plasmin-mediated cleavage of members of the complement system and immunoglobulin facilitates immune evasion of some strains of bacteria (22, 23). Bacteria also use plasminogen as a molecular linker to enable interaction with host cells (23).

Binding of plasminogen to *Cryptococcus neoformans* may facilitate the ability of this fungal pathogen to cross the blood brain barrier (24). It has been suggested that the affinity for plasminogen binding could reflect the observed strain differences in virulence of *C*. *neoformans* (24). However, plasminogen may not function in promoting virulence of all fungal pathogens. Although *Candida albicans* binds plasminogen and can cleave fibrin when in the presence of exogenous plasminogen activators, this binding does not affect virulence or endothelial damage and therefore the *in vivo* significance is not known (18). Multiple species of helminth parasites possess plasminogen binding proteins that facilitate their invasion and immune evasion (19). Protozoans are also considered to use plasminogen to support their host invasion but binding varies with morphotype and age for *Leishmania mexicana* (25). As not all pathogens can endogenously activate the zymogen they therefore require interaction with host plasminogen activators (18).

## Immune cells as sources of fibrinolytic proteins

Plasminogen activation is enhanced by assembly of plasminogen and its activators on fibrin or cellular surfaces (26–29) which also protect plasmin from inhibition by α_2_AP (30–32). Plasminogen receptors are found on endothelial cells, platelets, monocytes, macrophages and neutrophils [reviewed in (33)]. The multitude of plasminogen receptors have the common feature of availability of C-terminal basic residues (33). This includes binding proteins that lack a transmembrane protein (e.g., α-enolase and histone 2B), transmembrane proteins that require proteolysis to expose the C-terminal basic residue (e.g., integrins α_*IIb*_β_3_ and α_*M*_β_2_) and Plg-R_*KT*_ a transmembrane protein synthesized with a C-terminal lysine residue (33, 34).

Plg-R_*KT*_ was first identified on the surface of monocytes and macrophages and co-localizes with uPAR (35) and facilitates plasminogen activation by tPA (35) and uPA (36). Monocyte-derived uPA is required for incorporation of these cells into thrombi for efficient thrombus resolution (37). Although uPA is the predominant plasminogen activator in monocytes, stimulation with lipopolysaccharide (LPS), interferon-γ (IFN-γ) interleukin-4 (IL-4) all induce tPA secretion (38). Monocytes also express PAI-1 and are a major source of PAI-2 (39). Intracellular and secreted PAI-2 can be induced by stimulation of monocytes with thrombin and LPS (39, 40). Presence of PAI-2 in arterial and venous thrombi, presumed to be from monocytes, inhibits uPA-mediated lysis (41, 42). Both PAI-1 and PAI-2 are decreased by targeted upregulation of uPA which enhances fibrinolysis induced by monocyte-derived macrophages (43).

Thrombin activatable fibrinolysis inhibitor is also expressed by monocytes and macrophages with the level of expression being dependent on the activation status (44). Stimulation of macrophages with IL-4 downregulates TAFI expression whilst the proinflammatory stimuli IFN-γ and LPS has no effect (44). Additionally, monocytes and macrophages contain cellular FXIII-A (45, 46) which is trafficked to the membrane in association with golgi vesicles (47). IL-4 and IL-10-induced externalization of FXIII-A on monocytes stabilizes thrombi against degradation (48).

Polymorphonuclear leukocytes, assumed to be neutrophils, participate in endogenous thrombus lysis, mainly mediated by uPA with small contributions from tPA, elastase and cathepsin G (49). More recently neutrophils and their ability to form NETs have gained attention for their antifibrinolytic function. NETs consist of extruded nuclear and cytoplasmic content including histones, DNA strands and granular proteins including neutrophil elastase (4). The presence of DNA, histones and NETs inhibits plasminogen activation *in vitro* which can be reversed by degrading the chromatin with DNase (50, 51). Targeting DNA *in vivo* limits DVT growth in mice (52) and enhances tPA-mediated *ex vivo* thrombolysis of thrombi obtained from acute ischemic stroke patients (53, 54).

Alongside their role in promoting coagulation, platelets also regulate fibrinolysis and form part of the innate immune response. These anucleate cell fragments are packaged with granular content required for these multifaceted functions. Activated platelets expose P-selectin which facilitates interaction with the P-selectin glycoprotein ligand-1 (PSGL1) expressed on leukocytes and endothelial cells. Platelet-leukocyte interactions also occur via CD40-CD40L. These interactions allow platelets to direct leukocytes to sites of inflammation and propagate the inflammatory process (55, 56).

Platelet-rich thrombi are more resistant to lysis than erythrocyte-rich thrombi (57, 58) and platelets have largely been considered to be antifibrinolytic. Platelets are a major pool of circulating PAI-1 which is contained within the α-granules (59). Model thrombi formed at high shear rates contain elevated PAI-1 and lower tPA and plasminogen (60). This is consistent with the greater abundance of PAI-1 in platelet dense arterial thrombi compared to venous thrombi (61, 62). Platelet-derived PAI-1 is retained on activated platelet membranes, localizing to the platelet “cap” or “body” on phosphatidylserine (PS)-exposing procoagulant platelets or centrally over spread platelets (63, 64). This platelet-derived PAI-1 is functional in conferring resistance to lysis (63).

Additional anti-fibrinolytic factors contained within platelet α-granules include TAFI (65, 66), PN-1 (67), and α_2_AP (68, 69) which can downregulate fibrinolysis. The role of α_2_AP in maintaining thrombus stability may be limited as addition of circulating platelet concentration to α2AP-depeleted plasma does not protect against degradation (70). However, platelets contain a cytoplasmic pool of FXIII-A which crosslinks high molecular weight γ-dimers, α-polymers and α_2_AP-fibrin (71–75). Platelets retain externalized cellular FXIII-A in the “cap” region stabilizing thrombi against lysis due to crosslinking of α_2_AP (70). FXIII-A is also observed in platelet microparticles translocated via intracellular signaling that is calcium-independent (76).

In contrast to this, platelets support fibrinolytic activity through binding and exposure of plasminogen (28, 64, 77). Strong platelet stimulation facilitates plasminogen binding by fibrin-dependent and fibrin-independent mechanisms (64, 78). Plg-R_*KT*_ accounts for binding of approximately 40% platelet-derived plasminogen (28). Plasminogen activators also localize to the platelet surface with tPA binding being fibrinogen-dependent (65). Single chain uPA is activated on the platelet surface in a mechanism of reciprocal activation with plasminogen (77).

Platelet dense granules contain polyphosphate (polyP), a biomolecule which functions in modulation of coagulation and inflammation (79). PolyP delays fibrin polymerization altering clot structure (80). The knotted fibrin structure downregulates tPA and plasminogen binding thereby inhibiting tPA-mediated fibrinolysis (81). The effect on uPA-mediated plasminogen activation may depend on the contribution of other proteins as polyP accelerates activation in a purified system (82) whilst inhibits it in a plasma-based system (83). FXII has close structural homology to tPA and uPA and as such can function as a plasminogen activator. PolyP auto activates FXII to active single chain FXII (84) which facilitates plasminogen activation (85). Platelet-derive polyP could therefore have differential roles in thrombus resolution and cellular proteolytic process depending on the surrounding environment.

During vascular insult, many of the innate cell immune responses require interaction with the endothelium. Endothelial cells are the main source of circulating tPA, and secretion occurs via both constitutive and regulated mechanisms (86). Both plasminogen and tPA can bind to endothelial cells and therefore have the potential to generate plasmin (87). Endothelial cells also secrete uPA which bind to the cell surface uPAR (88). Additionally, endothelial cells produce the fibrinolytic inhibitors PAI-1 (89, 90), PAI-2 (91), and TAFI (92) which are upregulated in response to inflammatory cytokines.

Interaction of innate immune cells within the thrombus environment could influence resolution and stability. In pulmonary thrombi, rolling neutrophils rip membrane fragments from PS-exposing platelets facilitating formation of neutrophil macroaggregates (93). It is interesting to speculate that this could act to deliver platelet-derived fibrinolytic proteins within these aggregates and may facilitate platelet-neutrophil fibrinolytic crosstalk.

## The role of the fibrinolytic system in immunomodulation

Fibrinolytic proteins have a multitude of roles outside of their primary function of fibrin degradation including regulating the immune response. PAI-1 is an acute phase protein that is upregulated in response to injury, infection and inflammation (90, 94, 95) (Figure 1). Upregulation of PAI-1 is considered to be a protective mechanism important for early immune responses against bacterial pathogens, including *Haemophilus influenzae* (96), *Pseudomonas aeruginosa* (97). PAI-1 promotes bacterial clearance and limits inflammation (96). Downregulation of PAI-1 by *Streptococcus pneumoniae* pneumolysin is associated with increased mortality which can be reversed by administering recombinant PAI-1, protecting against alveolar haemorrhage (98).

Plasminogen activator inhibitor-1 facilitates neutrophil migration and its inhibition or deletion reduces influx at the site of injury in response to *Pseudomonas aeruginosa*, *Escherichia coli*, and *Klebsiella pneumoniae* infections (97, 99, 100). PAI-1 regulates IFN- γ in response to LPS and *Staphylococcal enterotoxin B* (101) in a mechanism independent of the plasminogen activators. PAI-1 may also have a protective role in viral infections, due to inhibition of proteases required for glycoprotein cleavage, therefore limiting viral replication (102).

TAFIa modulates inflammation by removal of C-terminal arginine or lysine residues from C3a, C5a, bradykinin osteopontin and chenerin (103–105) (Figure 1). Cleavage of C5a by TAFI is protective in inflammatory models of LPS induced acute lung injury (106), bronchial asthma (107), and rheumatoid arthritis (108). The development of post-traumatic sepsis is associated with a reduction in TAFI and increased C5a (109). Additionally, TAFI-deficient mice display enhanced neutrophil recruitment and tumor necrosis factor-α (TNF-α) and IL-6 levels in the peritoneum after *Escherichia coli* induced abdominal sepsis (110). This was independent of its antifibrinolytic function (110). In contrast to this, in *Pseudomonas aeruginosa*-induced sepsis, TAFI inhibition potentiates the effects of the antibiotic, ceftazidime and reduces organ dysfunction (111).

Plasmin(ogen) has multifaceted roles in the regulation of proinflammatory processes [reviewed in (20)]. Plasminogen is required for efficient recruitment of monocytes and lymphocytes in response to inflammation (112) and promotes macrophage phagocytosis and migration (113, 114) (Figure 1). Deficiency of plasminogen alters the expression of phagocytic genes (113). Whilst the fibrinolytic activity of plasmin is required for macrophage migration in experimental peritonitis (114). Interestingly, the absence of fibrinogen or the integrin α_*M*_β_2_ reverses the requirement for plasminogen suggesting fibrinolytic activity is required to remove the physical restraint of macrophages by fibrin(ogen) (114). Plg-R_KT,_ is upregulated during differentiation of monocytes to macrophages (35) and drives polarization to an M2-like macrophage phenotype (115). Additionally, dendritic cell phagocytosis is enhanced by plasmin which maintains these cells in an immature phenotype an reduces migration to the lymph nodes (116).

Plasminogen activators modulate the innate immune response, in mechanisms both dependent and independent of their fibrinolytic action. In a *Escherichia coli*-induced sepsis model, tPA deficiency caused increased bacterial loads, reduced neutrophil migration and was associated with increased mortality by a plasmin-independent mechanism (117) (Figure 1). Consistent with this, enzymatically inactive tPA blocks LPS induced increase in proinflammatory cytokines such as TNF-α, and IL-6 via low density lipoprotein receptor-related protein-1 (LRP1) and *N*-methyl-D-aspartic acid receptor (NMDA-R) (118, 119). However, in an ischemia/reperfusion model, tPA-mediated plasmin activity was required for neutrophil transmigration and disruption of endothelial junctions which allows further recruitment of neutrophils (120). Plasmin does not directly activate neutrophils and recruitment of these cells requires mast cell activation and leukotriene generation (120). Whilst in a stroke model, tPA-mediated plasmin generation decreased lymphocyte and monocyte counts, elevated IL-10 and TNF-α and altered splenic dendritic cell proportions (121).

Urokinase enhances monocyte differentiation into macrophages (122) and promotes neutrophil activation and migration (123). The uPA receptor, uPAR facilitates neutrophil migration in response to LPS-induced peritonitis, but this was not observed with *Escherichia coli* or in a polymicrobial sepsis model suggesting a compensatory mechanism may occur (124, 125). The function of uPAR on neutrophil migration is independent of its role in plasminogen activation and requires toll-like receptor signaling (125). Deficiency of uPAR promotes proinflammatory cytokines and macrophage polarization towards M1 phenotype and reduced phagocytosis in an experimental colitis model (126).

The varying roles of the fibrinolytic system in immunomodulation highlights the complex interactions which must be carefully balanced so as not exacerbate the inflammatory response and promote a prothrombotic environment.

## Dysregulation of fibrinolysis

Fibrinogen is an acute phase protein that dramatically increases during infection due to enhanced hepatic synthesis (127). Fibrin films form on the outside of blood clots which limit bacterial infiltration (128). However, aberrant fibrin accumulation contributes to development of a prothrombotic environment. During acute bacterial or viral infections, thrombotic complications can arise including deep vein thrombosis (DVT) and pulmonary embolisms (PE) (129, 130), acute myocardial infarction (AMI) (131, 132) and strokes (132). Thrombotic events occurring after infections affect various organ systems including respiratory, urinary and oral (133). The risk of thrombosis is higher in the first weeks succeeding infection and falls gradually after the initial infection (129). Consistent with a prothrombotic response to infection, seasonal variability in occurrence of AMI has been observed (134). The underlying mechanisms of the prothrombotic state are not fully understood. However, derailment of the fibrinolytic system is often a contributing factor to this.

Sepsis, a life-threatening response to infection, leads to tissue and organ damage and has a mortality rate of approximately 30%, although this is higher with older age or pre-existing conditions (135). As a result of the inflammatory state development of disseminated intravascular coagulation (DIC) can occur. This causes systemic dysregulation of coagulation and fibrinolysis resulting in depletion of coagulation factors and platelets and hemorrhaging. Platelet count is associated with severity, being significantly reduced with development of septic shock (136).

Plasmin(ogen) has a protective role in sepsis and levels are reduced with disease severity (137). However, a hypofibrinolytic state predominates in sepsis, largely due to elevated levels of PAI-1. Indeed, PAI-1 is a potential biomarker of disease severity and predictor of mortality (138). Initially, increased tPA and plasmin generation may predominate peaking at 2 h at which point TNF-α induces a steep increase in PAI-1 (139). Patients with the PAI-1 polymorphism 4G/5G, which is associated with elevated PAI-1 levels, are at increased risk of mortality from sepsis (140, 141). NETs may contribute to the elevated PAI-1 in sepsis as PAI-1 is downregulated in petidylarginine deiminase-4 (PAD-4) deficient mice which are unable to form NETs (142). NETs further contribute to a hypofibrinolytic state in sepsis due to the presence of cell-free DNA, an effect that can be overcome by DNase (143).

Hypofibrinolysis in sepsis may be further precipitated by other antifibrinolytic proteins. PAI-2 is not normally detected in healthy neutrophils but in patients with sepsis significant levels are present (144). Activation of TAFI could also be a contributing factor to the development of sepsis DIC (145). Interestingly, the TAFI Thr325 Ile/Ile single nucleotide polymorphism, which has increased antifibrinolytic potential, is associated with increased risk of contracting meningococcal disease and risk of mortality (146).

Acute respiratory distress syndrome (ARDS) is a hyperinflammatory condition that occurs in response to infection characterized by heightened alveolar-capillary permeability leading to extrusion of plasma proteins and inflammatory cytokines. This results in enhanced leukocytes and platelets recruitment to the lung microvasculature (147–149). Respiratory dysfunction and right heart failure develops, confounded by fibrin deposits which are observed in the air spaces and lung parenchyma due to the procoagulant environment along with hyaline-membranes and fibrosis (150–153).

Fibrin persistence is exacerbated by the inflammatory environment which promotes an imbalance in the fibrinolytic factors. Of note, PAI-1 synthesis is upregulated by several proinflammatory cytokines. Elevated levels of PAI-1 are observed with respiratory infections including influenza (154), severe acute respiratory syndrome coronavirus (SARS-CoV) (155) and SARS-CoV2 which downregulates fibrinolytic activity (156). Elevated PAI-1 is associated with worsening disease severity after SARS-CoV2 infection (156, 157). IL-6 induces an upregulation in PAI-1 gene expression and plasma levels of both PAI-1 and tPA (158–160). In endothelial cells, trans-signaling by IL-6 causes a circular amplification of IL-6 as well as IL-8, MCP-1 and PAI-1 synthesis (161). Additionally, endothelial cells release PAI-1 in response to the acute phase reactant, C-reactive protein (CRP) (150, 162, 163). Levels of uPA antigen are unaffected in ARDs but the heightened levels of PAI-1 cause a downregulation in fibrinolytic activity in the bronchoalveolar space (150).

## Therapeutic potential of targeting the fibrinolytic pathway

The appropriation of fibrinolytic system by pathogens to evade the host immune response and the varied function of fibrinolytic proteins in immunomodulation makes them potential therapeutic targets. Plasmin(ogen) binding and subsequent proteolytic activity are inhibited by lysine analogues. Lysine analogues therefore have potential in modulating the proinflammatory and immunosuppressive properties of plasmin. One such lysine analogue, epsilon aminocaproic acid (εACA), has been shown to reduce experimental Group B streptococcus meningitis and neonatal mortality rates (164). Whilst tranexamic acid (TXA), has shown promise at reducing rates of post-surgical infection (165). Furthermore, plasmin inhibition by aprotinin, εACA or TXA reduces neutrophil recruitment and may have potential to ischemia-reperfusion reduced injury (166).

On the other hand, when aberrant fibrin(ogen) develops during infection, promoting fibrinolysis is desirable. The use of recombinant tPA as an adjuvant therapy in a small retrospective study of infective endocarditis facilitated clearance of fibrin rich vegetations that encase the bacteria (167). In trauma or sepsis induced ARDS, uPA and streptokinase, were beneficial producing a significant improvement in PaO_2_ (168, 169). Coronavirus disease-19 (COVID-19) is caused by infection with severe acute respiratory SARS-CoV2. Severely ill patients with COVID-19 are prone to thrombosis and can develop ARDS, sepsis and multiorgan failure. Thrombolytic therapy has therefore garnered interest for treatment in severely ill COVID patients (170, 171). Initial studies indicate that tPA improves PaO_2_/FiO_2_ ratio, however, larger studies are required to establish treatment regimens and the safety profile (172, 173). Targeting the inflammatory response also has potential to correct fibrinolytic dysregulation. Indeed, blocking IL-6 with Tocilizumab decreases PAI-1 levels and this was found to be beneficial in SARS-CoV2 infection and is a recommended therapy in ICU patients (161, 174).

## Summary

The fibrinolytic and innate immune systems work in concert to protect from infection and inflammation and to regulate thrombus resolution. Derailment of one system therefore influences the other. Invading pathogens take advantage of plasminogen and its activators to evade protective immune responses. Whilst immune cells are a source of fibrinolytic proteins and act as a surface for their assembly and function in thrombus resolution. The fibrinolytic system participates in host immune responses, however, dysregulation can precipitate in aberrant fibrin distribution or impede immune cell function. There is much still to learn on the interplay between the fibrinolytic and innate immune systems. Improved understanding of these intricacies could lead to development of more targeted immunothrombolytic or immunomodulating therapies.

## Author contributions

The author confirms being the sole contributor of this work and has approved it for publication.
